# Effects of a combination of plant bioactive lipid compounds and biotin compared with monensin on body condition, energy metabolism and milk performance in transition dairy cows

**DOI:** 10.1371/journal.pone.0193685

**Published:** 2018-03-27

**Authors:** Janis Hausmann, Carolin Deiner, Amlan K. Patra, Irmgard Immig, Alexander Starke, Jörg R. Aschenbach

**Affiliations:** 1 Institute of Veterinary Physiology, Freie Universität Berlin, Berlin, Oertzenweg, Germany; 2 Department of Animal Nutrition, West Bengal University of Animal and Fishery Sciences, Belgachia, Kolkata, India; 3 DSM Nutritional Products Ltd., Kaiseraugst, Wurmisweg, Switzerland; 4 Clinic for Ruminants and Swine, University of Leipzig, Leipzig, Germany; University of Illinois, UNITED STATES

## Abstract

The aim of this study was to test whether a combination of plant bioactive lipid compounds (also termed ‘essential oils’) and biotin (PBLC+B) could decrease the mobilization of body reserves and ketosis incidence in postpartum dairy cows. We compared non-supplemented control (CON) cows with cows receiving monensin (MON) as a controlled-release capsule at d -21, and with cows receiving PBLC+B from day (d) -21 before calving until calving (Phase 1) and further until d 37 after calving (Phase 2), followed by PBLC+B discontinuation from d 38 to d 58 (Phase 3). The PBLC+B cows had higher body weight and higher back fat thickness than CON cows and lesser body weight change than MON and CON cows in Phase 3. Body condition score was not different among groups. Milk protein concentration tended to be higher on the first herd test day in PBLC+B vs. CON cows. Milk fat concentration tended to be highest in PBLC+B cows throughout Phases 2 and 3, with significantly higher values in PBLC+B vs. MON cows on the second herd test day. Yields of energy-corrected milk were higher in PBLC+B vs. CON and MON cows in Phase 2 and higher in PBLC+B and MON cows vs. CON cows in Phase 3. The incidence of subclinical ketosis was 83%, 61% and 50% in CON, PBLC+B and MON cows, respectively, with lower mean β-hydroxybutyrate values in MON than in PBLC+B cows in Phase 1 prepartum. The serum triglyceride concentration was higher in PBLC+B vs. CON cows on d 37. No differences were observed in serum glucose, urea, non-esterified fatty acids, cholesterol and bilirubin concentrations. Aspartate transaminase and γ-glutamyltranspeptidase but not glutamate dehydrogenase activities tended to be highest in MON and lowest in PBLC+B in Phase 2. We conclude that PBLC+B prevent body weight loss after parturition and are associated with similar ketosis incidence and partly higher yields of energy-corrected milk compared to MON supplementation of dairy cows.

## Introduction

High-yielding dairy cows regularly experience metabolic challenges in early lactation. Following calving, their energy demand rises greatly because of the onset of milk production and the metabolic priority of the mammary gland [1–3]. In addition to significant energy output, especially via milk fat, lactose production is challenging because the demand of glucose rises abruptly [4]. A shortage of glucose leads to instant hormonal adaption via an increasing glucagon:insulin ratio [5], one major consequence being the mobilization of large amounts of body fat [6–8]. The mobilized non-esterified fatty acids (NEFA) can serve as an energy fuel in tissues capable of β-oxidation. For tissues not capable of β-oxidation (e.g. neuronal tissues), the liver produces ketone bodies as a glucose-substituting energy fuel [1, 6]. Despite their roles as essential metabolic fuels, high concentrations of ketone bodies and NEFA lead to redox damage [9, 10] and are associated with production losses and health disturbances, commonly referred to as ketosis of transition dairy cows. Clinical ketosis is easily detectable based on clinical signs such as the abrupt loss of appetite, decreased milk performance and neuronal (shivering, apathy, disorientation, blindness, cramps) and gastrointestinal (decreased ruminal motility, constipation) signs [6, 11–13]. The more subtle forms of subclinical ketosis are not as easily detectable because the associated weight and production losses are not linearly related to the degree of lipomobilization and ketone body production. In serum, minimum ketone body thresholds that provide an acceptable specificity for the prediction of health disturbances and production losses on the individual cow level have mostly been identified as lying between 1.2 to 1.4 mmol/L β-hydroxybutyric acid (BHB) [14].

Subclinical ketosis is highly prevalent in transition dairy cows with typical herd incidences of between 40 to 60% [15, 16]. The costs attributable to associated diseases and production losses have recently been estimated at $289 per case of hyperketonemia in the US [17]. The reduction of ketosis incidence is therefore a key issue in current dairy herd management and dairy research. Because a glucose deficit is the ultimate trigger for ketone production, major prevention strategies include the provision of gluconeogenic precursors in the form of feed additives or the modulation of ruminal fermentation to increase ruminal propionate production [4]. Ionophore antibiotics, especially monensin (MON), are potent enhancer of ruminal propionate production [18–20] and are thus widely used in many countries around the time of calving for the prevention of ketosis [15, 21] and associated diseases [22]. However, their prophylactic use has low public acceptance in several countries and was, for example, banned in the EU in 2006 [23]. In 2013, the reintroduction of MON into the European market as the controlled-release capsule Kexxtone^®^ (Elanco, Indianapolis, IN, USA) for targeted metaphylactic use to prevent hyperketonemia and associated diseases in risk animals [24] re-initiated a general public debate about antibiotic use in farming, especially, in Germany [25]. Therefore, despite the proven efficacy of the MON controlled-release capsule in ketosis prevention [21, 26, 27], alternative methods of ketosis prevention is an urgent need. Plant bioactive lipid compounds (PBLC), especially those traditionally referred to as ‘essential oils’, could provide such alternatives based on their antimicrobial effects [28–30] with the potential of stimulating ruminal propionate fermentation [31, 32]. Biotin, on the other hand, is a cofactor of propionyl-CoA carboxylase and pyruvate carboxylase [33] and might thus support gluconeogenesis at the level of intermediate metabolism [34–36]. In a preceding study, we have observed improved ruminal propionate fermentation and decreased loss of body condition in postpartum dairy cows when supplemented with both PBLC and biotin [32], named PBLC+B hereafter. The aim of the present study has been to substantiate such beneficial effects of PBLC+B on body condition with an additional focus on energy metabolism and milk performance when compared with untreated control (CON) cows. In the extension of our previous study, our intension has also been to compare the effects of PBLC+B with those of a widely applied metaphylactic scheme involving the use of MON.

## Materials and methods

### Animals, housing, feeding and treatments

The study was performed on a commercial dairy farm in Lower Saxony (Germany) and was approved by the State Office of Lower Saxony for Consumer Protection and Food Safety (ref. 33.12-42502-04-14/1661). The farm housed a total of 140 Holstein dairy cows. The study included 58 multiparous cows. It began in November 2014 and lasted until September 2015. Each cow remained in the trial for 11 weeks, starting on day (d) -21 before the expected calving date until d 58 postpartum. Cows were fed a partial mixed ration (PMR) and concentrates based on the recommendations of the Society of Nutrition Physiology (GfE) [37], allowing for 5% orts. The concentrates consisted of a basal concentrate C1 that was provided in the dry and lactation periods as explained later and a protein-rich concentrate C2 that was provided in the lactation period only (Table 1).

**Table 1:** Ingredients, analyzed chemical composition and energy contents of partial mixed ration and concentrates used in close-up and lactation diets.

	Partial mixed ration^1^		Concentrate^2^		
	Close-up	Lactation	C1^3^	C1*PBLC+B	C2^4^
**Ingredients (as fed)**	**g/kg**	**g/kg**	**g/kg**	**g/kg**	**g/kg**
Grass silage	423	528			
Corn silage	423	407			
Straw	95	4			
Wheat		20	155	155	
Protein booster^5^	42	29			
Premix P1^6^	17				
Premix P2^7^		12			
Rape extraction meal			270	270	330
Soy extraction meal					330
Corn			180	178	330
Sugar beet molasses					10
Palm kernel expeller			250	250	
Molassed beet pulp			50	50	
Dried beet pulp			50	50	
Sugar beet molasses			15	15	
Glucose molasses			10	10	
Rapeseed			10	10	
Calcium carbonate			8	8	
Sodium chloride			1	1	
Premix P3^8^			1	1	
CRINA^®^ Ruminants^9^				2	
ROVIMIX Biotin^9^				0.04	
Dry matter (DM)	360 ± 11.6	358 ± 6.9	894 ± 5.2	893 ± 5.5	887 ± 4.2
**Chemical composition**	**g/kg DM**	**g/kg DM**	**g/kg DM**	**g/kg DM**	**g/kg DM**
Crude ash	90 ± 2.2	89 ± 2.8	60 ± 1.0	64 ± 0.6	59 ± 0.0
Crude protein	136 ± 4.9	143 ± 3.7	196 ± 2.6	202 ± 3.2	305 ± 5.6
Utilizable crude protein	138 ± 2.5	143 ± 3.7			
Crude fibre	250 ± 9.5	235 ± 8.7	111 ± 8.0	128 ± 3.9	86 ± 3.7
Crude fat	36 ± 1.0	42 ± 3.1	38 ± 1.7	34 ± 4.0	36 ± 1.5
Sugar	10 ± 3.7	13 ± 3.1	80 ± 3.5	85 ± 5.4	67 ± 3.8
Starch	109 ± 11.0	125 ± 11.5	283 ± 5.7	235 ± 1.5	297 ± 3.0
aNDFom^10^	467 ± 10.7	448 ± 8.7	308 ± 4.8	341 ± 0.9	191 ± 5.0
ADFom^10^	260 ± 9.1	244 ± 9.6	166 ± 5.8	203 ± 0.7	125 ± 4.4
ADL^10^	27 ± 0.8	24 ± 1.7	53 ± 1.2	73 ± 1.2	41 ± 0.7
NFC^10^	271 ± 9.4	279 ± 5.1	398 ± 8.4	359 ± 2.9	408 ± 1.7
**Energy**	**MJ/kg DM**	**MJ/kg DM**	**MJ/kg DM**	**MJ/kg DM**	**MJ/kg DM**
Metabolic energy	10.0 ± 0.2	10.4 ± 0.3	12.6 ± 0.1	12.3 ± 0.1	13.3 ± 0.0
Net energy for lactation	6.0 ± 0.1	6.3 ± 0.2	7.9 ± 0.0	7.5 ± 0.1	8.4 ± 0.0

^1^Samples were taken weekly and pooled over two months for chemical analyses; chemical analysis data are means ± SEM of four (close-up diet) to five analyses (lactation diet).

^2^Samples were taken weekly and pooled over two or four months for chemical analysis; chemical analysis data are means ± SEM of three analyses.

^3^Based on milk-performance feed TvP Exclusiv 18/7 (Trede & von Pein)

^4^Based on soybean meal:rapeseed meal:corn, 1:1:1 (Trede & von Pein, Dammfleth, Germany)

^5^Protein booster, Lacto36solo (Trede & von Pein) containing (per kg): 480 g rape extraction meal, 480 g steam-heated soy extraction meal, and 40 g sugar beet molasses

^6^Premix P1, SALVAmin Prenatal TR-40 (with SALVANA dairy vital complex) (SALVANA Tiernahrung GmbH, Sparrieshoop, Germany) containing (per kg): 299 g MgO_2_, 258 g Ca(H_2_PO_4_)_2_, 220 g NaCl, 60 g wheat, 47 g sugar beet pulp, 20 g sugar beet molasses, 10 g fruit pulp, vitamin and mineral mix (the latter containing 900,000 IU vitamin A, 200,000 IU vitamin D_3_, 10,000 mg vitamin E, 950 mg Cu as CuSO_4_×5H_2_O, 250 mg Cu as Cu_2_(OH)_3_Cl, 5,500 mg Zn as ZnO, 1000 mg Zn as ZnCl(OH)×H_2_O, 5,000 mg Mn as MgO_2_, 50 mg I as Ca(IO_3_)_2_, 35 mg Se as Na_2_SeO_3,_ 10 mg Se as rumen-protected Na_2_SeO_3_, 5 mg Se in organic form as *Saccharomyces cerevisae*, 50 mg Co as CoCO_3_, and 33 × 10^9^ KBE *Saccharomyces cerivisea*)

^7^Premix P2, SALVANA Rinderstolz 9848 GF 600 (SALVANA Tiernahrung GmbH) containing (per kg): 336 g RaPass, 334 g calcium salts of palm oil fatty acids, 87 g calcium carbonate, 86 g sodium chloride, 56 g monocalciumphosphat, 30 g magnesiumoxid, 12 g sugar beet molasses,190,000 IU vitamin A, 19,000 IU vitamin D_3_, 1,500 mg vitamin E, 288 mg Cu as CuSO_4_×5H_2_O, 72 mg Cu as Cu_2_(OH)_3_Cl, 1,100 mg Zn as ZnO, 300 mg Zn as ZnCl(OH)×H_2_O, 100 mg Zn as glycin zinc chelate hydrate, 1,140 mg Mn as MgO_2_, 14 mg I as Ca(IO_3_)_2_, 6 mg Se as Na_2_SeO_3,_ 4 mg Se as rumen-protected Na_2_SeO_3_, 9 mg Co as CoCO_3_×H_2_O, and 33 × 10^9^ KBE *Saccharomyces cerevisiae*

^8^Premix P3, Premix cow 0.1% 8399 (Trede & von Pein) containing (per kg): 938 g calcium carbonate, 7,500,000 IU vitamin A, 875,000 IU vitamin D_3_, 19 g Mn as MgO_3_, 0.1 g I as Ca(IO_3_)_2_, 0.4 g Se as Na_2_SeO_3_, 0.2 g Co as CoCO_3_×H_2_O, 40 g Zn as ZnO

^9^Analyzed concentrations in the final C1*PBLC+B concentrate were 1.03 g/kg PBLC and 0.034 g/kg biotin.

^10^aNDFom = Neutral detergent fiber corrected for residual ash and analyzed with amylase; ADFom = acid detergent fiber corrected for residual ash; ADL = acid detergent lignin; NFC = non-fibre carbohydrates [100 - (% aNDFom + % crude protein + % crude fat + % crude ash)].

Before the start of the study, cows were blocked according to their day of expected calving, average milk yield in previous lactations, number of parturition and sex of the first calf and then randomly assigned to treatment within block. Sex of first calf was used as a blocking criterion because it has an epigenetic link to milk yield [38]. The first treatment group received 2 g/d CRINA^®^ Ruminants and 40 mg/d ROVIMIX^®^ Biotin (both from DSM Nutritional Products Ltd, Kaiseraugst, Switzerland). Treatment PBLC+B was provided as a daily feed supplement contained in 1 kg pelleted concentrate C1, named C1*PBLC+B hereafter (Table 1). The 1 kg C1*PBLC+B was supplied to each cow individually as a single portion once a day from d -21 before expected calving until d 37 after calving. CRINA^®^ Ruminants is a proprietary mixture of PBLC containing thymol, eugenol, limonene and vanillin as the main bioactive ingredients. It is a listed and authorized feed additive (list for feed additives of the EU (Reg 1831/2003), FEMA (Flavor and Extracts Manufacturers Association) and the GRAS (Generally Recognised as Safe) database of the US Food and Drug Administration). The recommended PBLC dose for lactating dairy cows is 1 g/d. Biotin is recommended at doses of 20 mg/d for lactating dairy cows [39, 40]. The recommended dosages were doubled in the present study as these higher dosages had proven effective in our previous study [32]. Furthermore, PBLC have dose-dependent effects on ruminal fermentation [31] and performance [41] that extend beyond the currently recommended dose by the supplier. Higher doses of 40 mg/d biotin have been associated with marginal additional benefits for milk protein content [42] and beneficial effects in the treatment of sole ulcers [43]. Each cow of the second treatment group received a single MON controlled-release capsule (Kexxtone^®^) on d -21 by using a rumen bolus applicator. The controlled-release capsule is designed to release ~335 mg/d MON into the rumen over a period of 95 d according to the information deposited at the (European) Community Register of Veterinary Medicinal Products (http://ec.europa.eu/health/documents/community-register/html/v145.htm). A third group served as a control (CON) and received neither the PBLC+B feed supplement nor the MON rumen bolus.

From d -21 until parturition, cows remained in a separate barn with a straw-bedded pen. To increase comfort levels and to minimize ranking fights, the pen was subdivided into units of ~30 m^2^ in which up to three cows were kept at a time. The pen was equipped with a feeding fence in which the cows were fixed to receive their manually allocated concentrate portion at 09:30 h. The distance between cows was such that every cow only had access to its individual concentrate portion, i.e. 1 kg/d concentrate C1 for cows in groups CON and MON and 1 kg/d concentrate C1*PBLC+B for cows in group PBLC+B. Once cows had consumed their concentrate feeds, they were released from the feeding fence and the first half of PMR was served at 10:00 h, followed by the second half of PMR at 18:00 h. The dry matter (DM) allowance from PMR was 12 kg/d in the close-up period. The composition of the PMR is shown in Table 1.

After calving, cows were moved to a free stall barn equipped with 110 lying places for an average of 125 cows in milk. The barn area was divided into two milking units, each served by a Lely Astronaut A4 milking robot (Lely S.à.r.l., Maassluis, The Netherlands). The milking robot allowed up to 4 daily visits per cow; each visit included the weighing of cows, milking, and feeding of a concentrate aliquot. To assure the consumption of the daily PBLC+B dose by each individual cow, 1 kg/d concentrate C1 was supplied first during the first daily milking. This first kilogram of concentrate C1 was either not supplemented (CON and MON groups) or supplemented with PBLC+B (i.e. concentrate C1*PBLC+B for cows of the PBLC+B group). The remaining daily allocation of concentrate consisted of equal parts of C1 and C2 (Table 1). The total daily concentrate allowance started with 3 kg/d after calving, was increased incrementally by 100 to 250 g/d between d 1 and d 6 and was further increased by 250 g/d between d 7 to 25 to a final allowance of 8.5 kg/d that was maintained until the end of the study. The unused concentrate allowance was recorded in order to calculate concentrate intake. Postpartum lactating cows were fed PMR for lactating cows (Table 1) at a feedbunk twice daily at 09:00 and 17:00 h. The average DM allowance from PMR was 16.6 kg/d in the lactation period.

Health was monitored daily by visual inspection and by the assessment of data from the milking robot. In addition to concentrate intake, BW and milk performance, the data from the milking robot included a physical activity index and rumination time of each cow. For this purpose, the neck collar of each cow was equipped with a Lely Qwes-HR System (including acceleration sensor, rumination microphone, microprocessor and memory) from which the continuously logged data were downloaded during milking. Health disturbances were treated by the farm veterinarian and were recorded. Special attention was given to the occurrence of clinical ketosis. The latter was diagnosed based on the clinical signs listed in the Introduction and concurrently high serum BHB concentrations with a cautious interpretation of clinical signs when 1.4 ≤ BHB < 3.0 mmol/L and strict interpretation of clinical signs when BHB ≥ 3.0 mmol/L. Based on predefined exclusion criteria for severe or recurrent diseases, two cows of the CON group had to be excluded because of a displaced abomasum, one cow in the MON group because of multiple mastitis and two cows in the PBLC+B group because of a displaced abomasum or multiple mastitis. Thus, the finally analyzed group sizes were 17, 18 and 18 cows in the CON, PBLC+B and MON groups, respectively.

### Sampling

The sampling of blood and the assessment of back fat thickness (BFT) and body condition score (BCS) were always carried out at the same time of the day between 07:30 and 08:30. For practical reasons, sampling days were Mondays, Wednesdays and Fridays based on the expected (prepartum values) or real (postpartum values) calving dates of each individual cow. Target sampling days were d -21, -14, -7 before calving and d 2, 9, 16, 23, 30, 37, 44, 51, 58 after calving, this timing being achieved with a variation of ± 1 d after calving. However, as calving varied from the predicted date by several days, d -21 was defined as the day of the inclusion in the study and finally varied by ± 4 d to the real d -21. Day -7 was defined as the last sampling at least 4 d prior to real calving, i.e. d -7 ± 3. Day -14 was not available in some cows that calved earlier than expected and was thus removed from the data set.

### Blood sampling and analyses

The blood samples were collected from the coccygeal vein by using one 10-mL monovette with clot activator/additive carrier (S-Monovettes, Sarstedt, Nümbrecht, Germany). Within 1–2 h after taking the blood samples, serum was separated by centrifugation at 3000 *g* for 10 min (centrifuge Z364, BHG Hermle GmbH u Co., Gosheim, Germany) and stored at -20°C until analysis. The serum concentrations of glucose, BHB, NEFA, triglycerides, bilirubin, cholesterol and urea and the serum activities of aspartate transaminase (AST), γ-glutamyltranspeptidase (GGT) and glutamate dehydrogenase (GLDH) were measured by a Cobas C 311 (Roche Diagnostics Deutschland GmbH, Mannheim, Germany) in the Laboratory of the Clinic for Internal Veterinary Medicine of Leipzig University (Leipzig, Germany) with ready-to-use *e* pack reagents (Roche Diagnostics Deutschland GmbH).

### Body condition score and back fat thickness

The BCS was evaluated on a scale from 1 (emaciated) to 5 (overweight) in increments of 0.25 according to Edmonson et al. [44] with the only modification being that the transverse processes were assessed by using both visual and tactile assessment. The BCS was always assessed by the same trained person. The BFT was measured by ultrasound with an Eickemeyer Magic 1100 Smart Scanner equipped with a linear transducer as recommended by Staufenbiel [45]. The measuring point laid on the connecting line between the upper part of the Tuber ischiadicum and the upper part of the Tuber coxae, at one hand width cranial of the Tuber ischiadicum. The measurement included the skin and subcutaneous fat up to the Fascia trunci profunda. Ethanol (70%; Carl Roth GmbH u Co. KG, Karlsruhe, Germany) was used as a coupling medium on non-depilated skin [45].

### Body weight (BW)

The average body weight per cow per day was automatically calculated daily by the milking robot software by using the body weight recorded before each milking, which was up to four times per day.

### Milk yield and milk components

The milk yield was recorded by the milking robot at each milking and was summed by the software of the milking robot per cow per day. Milk components were determined in the monthly herd test by the MKV EW (Milchkontrollverband Elbe-Weser e.V., Hankensbüttel, Germany), which was performed if cows were ≥ 5 DIM. Milk sample collection on test days included all milkings in a 24-h sampling period by using the ICAR-approved Lely Shuttle milk sampling unit (Lely S.à.r.l.). The concentrations of fat, crude protein and urea were analyzed by infrared spectrophotometry and somatic cell count by flow cytometry using Combi Foss (Foss Electric, Hillerød, Denmark). In addition to the data from the milk test days, the milking robot was equipped with flow velocity, conductivity and optical sensors to estimate the daily milk fat and protein percentages according to proprietary algorithms. The latter data were used to extrapolate the daily energy-corrected milk yield (ECM). The ECM was calculated using the formula of German herd testing organizations (LKV) extrapolating to 40 g/kg fat and 34 g/kg crude protein (equivalent to 32 g/kg true protein): ECM (kg/d) = milk yield (kg/d) × [0.038 × fat (g/kg) + 0.021 × protein (g/kg) + 1.05] / 3.28.

### Feed analysis

Samples of PMR were taken weekly directly after feed supply at 5–10 different places on the feeding table. The samples were stored in labelled freezer bags at -20°C and pooled over two months before analysis. Samples of concentrates were taken weekly from the concentrate silos, stored in labelled freezer bags at -20°C and pooled over two to four months before analysis. Feed analyses were performed in the laboratory of the LKS Landwirtschaftliche Kommunikations und Service GmbH (Lichtenwalde, Germany) according to standard procedures [46]. Dry matter was determined in a drying cabinet (VDLUFA MB III 3.1). For concentrates, crude ash was determined in a muffle furnace at 550°C (VDLUFA III 8.1). Starch was determined by using a polarimeter (VDLUFA MB III 7.2.1), crude fat by the Soxhlet method with hydrolysis (VDLUFA MB III 5.1.1) and crude protein by incineration (VDLUFA MB III 4.1.2). Acid detergent fibre expressed exclusive of residual ash (ADF_om_) was determined by using Fibertec^TM^ 8000 (FOSS, Hilleroed, Denmark; method, VDLUFA MB III 6.5.2). Neutral detergent fibre (comparable to assay with heat-stable amylase and expressed exclusive of residual ash; aNDF_om_), sugar, acid detergent lignin (ADL) and chemical components of PMR were estimated by near-infrared spectroscopy according to the method VDLUFA MB III 31.2. Gas formation for the estimation of net energy for lactation in feeds was measured by using the Hohenheim Gas Test (VDLUFA MB III 25.1).

### Statistical analysis

Statistical analyses were performed by using the software SAS (2001; version 8.2, SAS Institute Inc, Cary, USA) and Sigma Plot 11.0 (Systat Software GmbH, Erkrath, Germany). Before all analyses, data retrieved from the milking robot (BW, milk yield, milk composition and concentrate intake contained in Table 2, S1 Table, S2 Table, S1 Fig and S2 Fig) were pooled over three consecutive days; e.g. values for d 2 represent the arithmetic mean of d 1 to 3. Data were allocated to three experimental phases: the prepartum Phase 1 including d -21 to d -1, the PBLC+B supplementation Phase 2 from d 1 to d 37, and the post-supplementation Phase 3 from d 38 to d 58. Data were analyzed as a randomised block design using the mixed model procedure of SAS with repeated measures within a phase. The model contained group, parity as block, day as the repeated measure with cow as subject, and interaction between group and day as the main effects. The phases were distinct periods with respect to physiological stages and dietary treatments, which were usually significantly varied for different response variables in this study. Parity was excluded from the model, if it increased *P*-values. Random effect of cow was included in the model, if it improved model fit according to Akaike (or Bayesian) information criterion (AIC). Different covariance structures including compound symmetry (type = cs), autoregressive (type = ar(1)), unstructured (type = un), variance components (type = vc) and Toeplitz (type = toep) were evaluated and the best covariance structure was finally selected according to the best model fit (smaller-is-better rule of the AIC values) and lower *P*-values. Usually, statistical models containing random effect of cow, parity and autoregressive covariance structure showed better model fit. When the interaction between group and day was significant (*P* < 0.05), the ‘slice’ option in the ‘lsmeans’ statement was used to determine differences among the treatments at each day. Comparisons among overall groups or groups at a day (if they were found to be significant based on the ‘slice’ option) were performed using ‘diff’ option in the ‘lsmeans’ statement.

**Table 2:** Concentrate intake, body weight (BW), BW changes, body condition score and back fat thickness of cows receiving plant bioactive lipid compounds and biotin (PBLC+B; n = 18) from d -21 to 37 relative to parturition, cows receiving a monensin bolus (MON; n = 18) at d -21 or cows receiving no such supplements (CON; n = 17).

Item	Group^1^			SEM	*P*-values		
	CON	PBLC+B	MON		Group	Day	Group × day
**Phase 1**							
Body condition score	3.56	3.74	3.63	0.141	0.41	<0.001	0.15
Back fat thickness, cm	2.86	3.33	3.21	0.268	0.37	0.34	0.23
**Phase 2**							
Concentrate intake, kg/d^2^	5.01	4.98	4.85	0.344	0.90	<0.001	0.003
Body condition score	2.89	3.09	2.99	0.100	0.26	<0.001	0.88
Back fat thickness, cm	1.96	2.50	2.27	0.197	0.099	<0.001	0.80
BW, kg	685^b^	746^a^	723^ab^	22.8	0.049	<0.001	0.39
BW change, kg	-45	-19	-46	14.0	0.089	<0.001	0.48
**Phase 3**							
Concentrate intake, kg/d	6.94	6.77	7.14	0.381	0.70	0.041	0.13
Body condition score	2.42	2.64	2.58	0.091	0.17	<0.001	0.73
Back fat thickness, cm	1.18^b^	1.65^a^	1.51^ab^	0.145	0.048	<0.001	0.81
BW, kg	663^b^	750^a^	689^ab^	24.6	<0.001	0.57	0.67
BW change, kg	-72^b^	-19^a^	-65^b^	14.9	0.002	<0.001	0.92

Phase 1: from d -21 to d -1 relative to parturition; Phase 2: from d 1 to d 37 relative to parturition; Phase 3: from d 38 to d 58 relative to parturition. SEM = standard error of mean (for group × day).

^1^Least square mean values of the groups are reported.

^2^For concentrate intake, diet effect was not significant at any individual day.

^ab^Superscript letters indicate differences among treatment groups at *P* < 0.05.

Data from individual days (Table 3 and all supporting material) were analyzed by Sigma Plot 11.0 (Systat Software GmbH, Erkrath, Germany) and are expressed as means and pooled SEM. Before statistical comparisons, data sets were tested for normality (Kolmogorov-Smirnov’s test with Lilliefors’ correction) and equal variance (Levene’s median test). If both tests were passed, data at each time point were compared by one-way ANOVA. If either test failed, the Kruskal-Wallis one-way ANOVA on ranks was performed. The multiple comparison procedure of Student-Newman-Keuls was performed to identify the means that differed.

**Table 3:** Milk performance on herd test days of cows receiving plant bioactive lipid compounds and biotin (PBLC+B) from d -21 to 37 relative to parturition, cows receiving a monensin bolus (MON) at d -21 or cows receiving no such supplements (CON).

Item		First test day					Second test day			
	CON	PBLC+B	MON	SEM	*P*-value	CON	PBLC+B	MON	SEM	*P*-value
Days after calving	22	20	21	4.4	0.55	54	52	53	5.2	0.86
Milk yield, kg/d	39.1	39.0	41.1	3.06	0.72	42.0	45.0	46.3	2.38	0.17
ECM yield, kg/d	40.4	41.5	42.2	2.67	0.52	40.6	44.4	42.7	2.31	0.13
Fat, %	4.59	4.64	4.53	0.276	0.93	3.97^ab^	4.09^a^	3.56^b^	0.160	0.004
Protein, %	3.11	3.32	3.18	0.092	0.076	2.98	3.04	2.98	0.066	0.53
Urea, mg/L	240	236	267	19.3	0.17	254	261	266	15.7	0.74
Somatic cell count, cells/L	401^1^	97	61	34.5	0.83	741^1^	124	169	71.2	0.45

Data are presented as means and pooled SEM of 17 cows in the CON group, 18 cows in the PBLC+B group and 18 cows in the MON group.

^ab^Superscript letters indicate differences among treatment groups at *P* < 0.05.

^1^The CON group included two cows with >1000 somatic cells/L at each test day

ECM = energy corrected milk; SEM = standard error of mean

Significant differences were accepted at *P* < 0.05. Trends were generally considered when 0.05 *< P <* 0.10. However, trends for effects on milk and ECM yields were considered when 0.05 *< P <* 0.15 because these variables appeared far outside the power range of the test design.

## Results

### General health records

No obvious differences were found in the clinical health status among groups. Clinical ketosis was not observed (for subclinical ketosis, see later section). Postpartum diseases for all 58 cows that initially entered the study included retained placenta (MON, n = 2), metritis (CON, n = 5; PBLC+B, n = 2; MON, n = 3), mastitis (CON, n = 4; PBLC+B, n = 6; MON, n = 6), lameness (CON, n = 4; PBLC+B, n = 4; MON, n = 2) and displaced abomasum (CON, n = 2; PBLC+B, n = 1). Some cows were given calcium (CON, n = 1; PBLC+B, n = 4; MON, n = 5) and/or phosphorous infusions (CON, n = 2; PBLC+B, n = 1). Treatments and calcium and phosphorous infusions were applied by the local veterinarian and cows generally responded well. However, five of the cows listed here had to be excluded from further analyses based on pre-defined exclusion criteria, including displaced abomasum and recurring mastitis (CON, n = 2; PBLC+B, n = 2; MON; n = 1).

### Concentrate intakes

The concentrate intakes resulting from the concentrate allowance minus unused concentrate allowance are listed in Table 2 for Phase 2 and Phase 3, and in S1 Table for individual days. Day effect was significant (*P* < 0.05; Table 2) as expected. Group × day interaction was significant in Phase 2 (*P* < 0.05; Table 2); however, no differences in concentrate intakes were noted among groups at any specific day (S1 Table).

### Body condition

Body condition was assessed based on BFT and BCS recorded weekly by the same operator, whereas BW was recorded daily by the milking robot after the beginning of lactation and pooled over three consecutive days. The BFT was not different in Phase 1 prepartum, but PBLC+B cows showed a trend for values higher than CON in postpartal Phase 2 (*P* = 0.099) and significantly higher values in Phase 3 (*P* = 0.048), with MON cows being intermediate. Similarly, although least square mean (LSM) of BCS were not different for the three phases (Table 2), BCS of PBLC+B cows showed a trend for values higher than CON at several individual days after parturition (d 9, 16, 23, 51 and 58; *P* < 0.1) with significantly higher values than CON at d 44 (*P* < 0.05; S2 Table). A very clear effect was seen on BW. Cows of the PBLC+B group had higher BW than CON in both Phase 2 and Phase 3 (*P* < 0.05; Table 2). When plotting the BW change relative to d 2 after parturition, it became obvious that cows of the PBLC+B group almost retained their parturition BW throughout the postpartum period in contrast to CON and MON cows that showed a trend for higher BW loss in Phase 2 (*P* = 0.089) and significantly higher BW loss in Phase 3 (*P* = 0.002; Table 2; S1 Fig).

### Milk yield and milk components

The first and second herd tests were performed 20 to 22 (± 4.4) and 52 to 54 (± 5.2) d after calving, respectively, with no differences among groups (Table 3). Daily milk yield was not different among groups on these days (Table 3). On the first herd test day, the milk fat concentration was not different among groups; however, a trend for higher milk protein concentration was observed in the PBLC+B group compared with the other two groups (*P* < 0.1). The trend for higher milk protein concentration did not continue until the second test day. Instead, milk fat concentration was higher on the second test day in PBLC+B vs. MON cows (*P* < 0.01), with CON cows showing intermediate values. Concomitantly, ECM yield showed a trend for higher values in PBLC+B vs. CON cows (*P* = 0.13; Table 3). Milk urea concentration and somatic cell count were not different among groups on the herd test days.

Data from the milking robot are presented in Table 4 and, for individual days, in the supporting material (S1 Table and S2 Fig). Frequency of milk robot visits did not differ among groups throughout the trial period (Table 4; S1 Table). Daily milk yield was not different among groups in Phase 2 but was higher in MON vs. CON cows in Phase 2 (*P* = 0.001), with PBLC+B cows showing intermediate values. The yield of ECM was higher in PBLC+B cows compared to both CON and MON cows in Phase 2 (*P* < 0.001; Table 4). In Phase 3, ECM yield was higher in PLBLC+B and MON cows compared with CON cows (*P* < 0.001; Table 4). The higher yields of ECM in the PBLC+B group was based, in part, on a trend for higher estimated milk fat percentage of cows of the PBLC+B group compared to both other groups in Phase 2 and Phase 3 (*P* < 0.1), coinciding with a trend for higher milk fat yield in Phase 2 (*P* = 0.094; Table 4). An interaction between group × day (*P* = 0.01) indicated that estimated milk fat percentage of PBLC+B cows was higher than that of CON cows at the beginning of Phase 2 (d 5 and d 8), higher than that of MON cows in the middle of Phase 2 (d 20 and d 26) and higher than that of MON and CON cows at the end of Phase 2 (d 35; *P* < 0.05; Table 4; S2 Fig). The estimated milk protein percentage and yield showed no difference among groups (Table 4), except for individual d 2, when estimated milk protein percentage was higher in PBLC+B cows compared with CON and MON cows (*P* < 0.05; S2 Fig).

**Table 4:** Milk robot visits, milk yield and milk composition of cows receiving plant bioactive lipid compounds and biotin (PBLC+B; n = 18) from d -21 to d 37 relative to parturition, cows receiving a monensin bolus (MON; n = 18) at d -21 or cows receiving no such supplements (CON; n = 17).

Item	Group^1^			SEM	*P*-values		
	CON	PBLC+B	MON		Group	Day	Group × day
**Phase 2**							
Milk robot visit per day	2.7	2.7	2.8	0.20	0.75	<0.001	0.17
Milk yield, kg/d	36.2	37.3	37.6	1.12	0.063	<0.001	0.99
ECM yield, kg/d	36.8^b^	39.5^a^	37.6^b^	0.48	<0.001	<0.001	1.00
Milk fat, %^2^	4.16	4.48	4.10	0.161	0.083	<0.001	0.010
Milk protein, %	3.55	3.63	3.55	0.060	0.35	<0.001	0.13
Fat yield, kg/d	1.55	1.74	1.58	0.081	0.094	0.003	0.99
Protein yield, kg/d	1.26	1.31	1.29	0.060	0.68	<0.001	0.84
**Phase 3**							
Milk robot visit per day	3.1	3.2	3.1	0.21	0.89	0.86	0.83
yield, kg/d	43.6^b^	45.0^ab^	46.6^a^	1.10	0.001	1.00	1.00
ECM yield, kg/d	41.6^b^	45.0^a^	44.1^a^	0.63	<0.001	1.00	1.00
Milk fat, %	3.69	4.03	3.66	0.135	0.055	0.33	0.47
Milk protein, %	3.26	3.32	3.23	0.053	0.43	0.68	0.47
Fat yield, kg/d	1.61	1.82	1.69	0.079	0.12	0.97	0.89
Protein yield, kg/d	1.43	1.49	1.50	0.055	0.49	0.82	0.43

Phase 2: from d 1 to d 37 relative to parturition; Phase 3: from d 38 to d 58 relative to parturition. ECM = energy corrected milk; SEM = standard error of mean (for group × day).

^1^Least square mean values of the groups are reported.

^2^For milk fat percentage: at d 5 and d 8, CON vs. PBLC+B: *P* < 0.05; at d 20 and d 26, MON vs. PBLC+B: *P* < 0.05; at d 35, CON vs. PBLC and MON vs. PBLC+B: *P* < 0.05.

^ab^Superscript letters indicate differences among treatment groups at *P* < 0.05.

### Blood serum metabolites and enzymes

Key indicators of energy metabolism are shown in Table 5 for phases and in S3 Table for individual days. Serum glucose concentrations generally decreased with progressing of trial days in Phase 1 and Phase 2 (*P* < 0.01) with no differences among groups and with no interaction effect between group and day in each of the three trial phases (Table 5). Serum NEFA values peaked at d 2 after parturition followed by a progressive decrease throughout Phases 2 and 3 (*P* < 0.01; Table 5; S3 Table), The peak in serum NEFA concentrations was followed by a peak in BHB concentrations between 16 d to 30 d after parturition as indicated by day effects in Phase 1 and Phase 2 (*P* < 0.01; Table 5; S3 Table). One prominent finding was a lower BHB value in MON cows compared with PBLC+B cows in Phase 1 before parturition, with CON cows having intermediate values (*P* < 0.05; Table 5). A trend for a group × day interaction indicated that this was primarily due to lower BHB values in MON vs. CON and PBLC+B at d -7 (*P* < 0.01; S3 Table). Based on a cut-off value of 1.4 mmol/L, the incidence of ketosis was 83% in CON, 61% in PBLC+B and 50% in MON cows over the whole trial period. Serum NEFA concentrations did not differ among groups (Table 5). The triglyceride concentration generally decreased after parturition in Phase 2 in all groups (*P* < 0.001). However, a group × day interaction (*P* = 0.033) indicated that serum triglyceride concentrations were higher in the PBLC+B group compared with the CON group on d 37 (Table 5; S3 Table).

**Table 5:** Serum metabolite concentrations in cows receiving plant bioactive lipid compounds and biotin (PBLC+B; n = 18) from d -21 to 37 relative to parturition, cows receiving a monensin bolus (MON; n = 18) at d -21 or cows receiving no such supplements (CON; n = 17).

Item	Group^1^			SEM	*P*-values		
	CON	PBLC+B	MON		Group	Day	Group × day
**Phase 1**							
Glucose, mmol/L	3.70	3.64	3.72	0.077	0.72	0.003	0.86
BHB, mmol/L	0.77^ab^	0.83^a^	0.68^b^	0.047	0.024	0.004	0.097
NEFA, mmol/L	0.15	0.17	0.20	0.035	0.40	<0.001	0.54
Triglycerides, mmol/L	0.29	0.31	0.28	0.018	0.35	0.10	0.39
Cholesterol, mmol/L	2.38	2.39	2.33	0.167	0.96	<0.001	0.98
Bilirubin, μmol/L	1.16	1.06	1.30	0.278	0.72	<0.001	0.65
Urea, mmol/L	4.17	4.32	4.35	0.187	0.66	0.20	0.27
**Phase 2**							
Glucose, mmol/L	2.99	2.97	3.18	0.143	0.17	<0.001	0.34
BHB, mmol/L	1.64	1.48	1.33	0.183	0.28	<0.001	0.23
NEFA, mmol/L	0.45	0.48	0.43	0.059	0.36	<0.001	0.78
Triglycerides, mmol/L^2^	0.13	0.14	0.13	0.006	0.52	<0.001	0.033
Cholesterol, mmol/L^3^	2.82	2.98	2.90	0.185	0.78	<0.001	0.007
Bilirubin, μmol/L	2.81	2.13	2.94	0.519	0.18	<0.001	0.32
Urea, mmol/L	4.41	4.25	4.68	0.244	0.16	<0.001	0.82
**Phase 3**							
Glucose, mmol/L	3.01	3.15	3.10	0.114	0.55	0.46	0.37
BHB, mmol/L	1.61	1.31	1.31	0.218	0.47	0.11	0.48
NEFA, mmol/L	0.19	0.20	0.21	0.023	0.78	0.001	0.65
Triglycerides, mmol/L	0.15	0.17	0.17	0.009	0.24	0.16	0.50
Cholesterol, mmol/L	4.48	4.78	4.80	0.271	0.60	<0.001	0.76
Bilirubin, μmol/L	0.73	0.82	0.98	0.157	0.28	0.37	0.12
Urea, mmol/L	5.07	4.86	5.14	0.226	0.52	0.014	0.47

Phase 1: from d -21 to d -1 relative to parturition; Phase 2: from d 1 to d 37 relative to parturition; Phase 3: from d 38 to d 58 relative to parturition. BHB = β-hydroxybutyric acid; NEFA = non-esterified fatty acids; SEM = standard error of mean (for group × day).

^1^Least square mean values of the groups are reported.

^2^For triglyceride at d 37, CON vs. PBLC+B: *P* = 0.005.

^3^For cholesterol, treatment effect was not significant at any individual day.

^ab^Superscript letters indicate differences among treatment groups at *P* < 0.05.

Other metabolic indicators (cholesterol, bilirubin and urea; Table 5; S4 Table) and serum activities of enzymes with relation to liver and muscle function (AST, GGT and GLDH; Table 6; S5 Table) showed no differences among groups (*P* > 0.1), except for a trend for higher AST and GGT values in MON in Phase 2 (*P* < 0.1; Table 6). Day effects were noted for most of these variables in different phases. With advancing time of Phase 1, concentrations of cholesterol and the activity of GGT generally decreased (*P* < 0.001); whereas, concentrations of bilirubin increased (*P* < 0.001). In Phase 2, the activity of AST decreased gradually (*P* < 0.001); whereas concentrations of cholesterol, bilirubin and urea, as well as the activities of GGT and GLDH, increased (*P* < 0.001). In Phase 3, the activity of GGT decreased (*P* = 0.045); whereas concentrations of cholesterol (*P* < 0.001) and urea (*P* = 0.014) increased (Tables 5 and 6; S4 and S5 Tables).

**Table 6:** Serum activities of enzymes in cows receiving plant bioactive lipid compounds and biotin (PBLC+B; n = 18) from d -21 to 37 relative to parturition, cows receiving a monensin bolus (MON; n = 18) at d -21 or cows receiving no such supplements (CON; n = 17).

Item	Group^1^			SEM	*P*-values		
	CON	PBLC+B	MON		Group	Day	Group × day
**Phase 1**							
AST, U/L^2^	67.4	67.2	79.7	5.66	0.16	0.33	0.045
GGT, U/L	23.6	24.8	25.7	1.74	0.65	<0.001	0.96
GLDH, U/L	11.0	11.2	12.5	2.63	0.88	0.12	0.088
**Phase 2**							
AST, U/L	98.3	89.3	109.8	9.23	0.094	<0.001	0.55
GGT, U/L	29.1	27.5	33.9	2.15	0.093	<0.001	0.76
GLDH, U/L	18.7	16.1	20.3	6.26	0.79	<0.001	0.94
**Phase 3**							
AST, U/L	80.9	70.9	83.9	5.53	0.107	0.59	0.48
GGT, U/L	30.7	30.4	36.2	2.64	0.16	0.045	0.49
GLDH, U/L	16.5	13.8	16.0	4.20	0.85	0.53	0.17

Phase 1: from d -21 to d -1 relative to parturition; Phase 2: from d 1 to d 37 relative to parturition; Phase 3: from d 38 to d 58 relative to parturition. AST = Aspartate transaminase; GGT = γ-Glutamyl transpeptidase, U/L; GLDH = Glutamate dehydrogenase, U/L; SEM = standard error of mean (for group × day).

^1^Least square mean values of the groups are reported.

^2^For AST at d -7, CON vs. MON: *P* = 0.033 and PBLC+B vs. MON: *P* = 0.022.

One outlier for GLDH was removed in MON.

## Discussion

To date, several nutritional intervention strategies have been developed to optimize energy metabolism around parturition and, thereby, to decrease the incidence of subclinical ketosis of dairy cows. As the pathophysiology of ketosis proceeds from a shortage of energy, especially in the form of glucose [4, 47], most strategies target measures to increase energy density in the diet and energy intake [48–50], measures to increase the supply of glucogenic precursors and measures to enhance the conversion of glucogenic precursors to glucose [51–53].

In the present study, we used MON as one current ‘gold standard’ supplement that targets ketosis prevention after calving [21, 54, 55]. The main action of MON is to increase ruminal propionate production, thereby, increasing the supply of this glucogenic precursor [20, 21, 56]. Previous trials have shown that PBLC can equally increase ruminal propionate production [31, 32, 56] and, when combined with biotin as a cofactor of gluconeogenic enzymes (propionyl-CoA carboxylase and pyruvate carboxylase; [33]), it can possibly provide additional benefits for body condition [32].

To compare the effects of PBLC+B with those of MON, we performed a controlled study in a commercial dairy herd that was managed according to very high standards but that nevertheless had a history of frequent cases of subclinical ketosis. We included all multiparous cows that calved in the study period, because multiparous cows are especially prone to metabolic dysfunctions in early lactation [57]. The design of the present study involved labour- and cost-intensive in-depth investigations in a representative number of individual cows. As such, the number of included cows did not allow a statistical comparison of general health benefits among treatments. At a descriptive level, however, no obvious differences were seen among the groups.

No clinical ketosis was observed during the course of the study. As expected from the herd history, however, subclinical ketosis was highly prevalent throughout of the study, especially, in the CON group. The cut-off points at which serum BHB concentrations of individual cows are verifiably associated with diseases have mostly been identified between 1.2 and 1.4 mmol/L [14]. Even when applying the higher value of 1.4 mmol/L, the incidence of subclinical ketosis was 83% in the CON group. Ketosis incidence in the PCLB+B and MON groups was 61% and 50%, respectively, with MON additionally inducing a decrease in mean serum concentrations of BHB around calving. This is in agreement with the previously established potential of MON to decrease the incidence of subclinical ketosis in postpartum dairy cows [21, 54, 55] and furthermore indicates a similar potential for the tested combination of PBLC+B.

Serum variables other than BHB were either not or not markedly altered by the two treatments. Serum glucose concentrations were generally very low in all groups, especially immediately after calving. This was as expected, because the shortage of glucose is the ultimate trigger of ketone body production in the liver. The latter was recently substantiated by Ruoff et al. [58] who identified that hypoglycaemia after calving is associated with the onset of hyperketonaemia in multiparous cows. When analysing individual days, occasional trends were noted for higher serum glucose concentrations in the PBLC+B and MON groups and for lower urea concentrations in the PBLC+B group. However, because the group and group × day *P*-values for these variables were > 0.1, care should be taken to interpret this as indicators of improved glucose and protein metabolism, respectively, especially when considering that other factors can also have an impact on these metabolites. Serum triglyceride concentration was slightly higher in the PBLC+B group on d 37, which may have some relationship to the higher milk fat concentrations observed in this group (see later). On the other hand, serum concentrations of NEFA, cholesterol and bilirubin were not different among groups.

The activity of the serum enzyme GLDH was also not different among groups. However, AST and GGT activities tended to be highest in the MON and lowest in the PBLC+B cows between 1 and 37 d after calving. Thus, our results are in partial contrast to a recent study involving the same MON controlled-release capsule and the same PBLC (at 1 g/d without biotin) in cows that were slightly more overconditioned (BCS = 3.95 ± 0.08) than the cows of the present study. In the previous study, GGT and GLDH activities selectively increased in the PBLC group during d 15 to d 56 after calving, indicating some degree of liver injury, whereas MON cows appeared to be protected [59]. In the present study, by contrast, the reference range of the analysing laboratory for AST (< 50 U/L), GGT (< 50 U/L) and GLDH (< 30 U/L) was exceeded most frequently in the MON group, and group values of AST and GGT activities tended to be lower in PBLC+B cows in Phase 1. Therefore, future studies should determine whether the increased GGT and GLDH activities observed by Drong et al. [59] after PBLC supplementation were merely an incidental finding or whether the co-supplementation of biotin should be routinely recommended to achieve optimum PBLC effects without compromising the liver, at least in overconditioned cows.

The most striking finding of the present study was the effect of PBLC+B on BW. Because of a negative energy balance, high-yielding dairy cows typically lose between 40 and 70 kg BW at one to two months after calving [60–62]. A weight loss of ~70 kg was also observed in the CON and MON groups in Phase 3 of the present study. The cows of the PBLC+B group, however, did not lose any significant weight and were the only group that had completely retained their BW at the end of the study. The prevented weight loss after calving in the PBLC+B group was partially reflected by a smaller loss of BFT; whereas, BCS points were not different for the analyzed phases but only some individual time points. Therefore, the reason for the protection against BW loss is not easily evident and probably includes any combination of lower mobilization of body fat, lower mobilization of lean body mass and/or increased feed intake, i.e. higher weight of the gastrointestinal tract. An argument in favour of an PBLC+B effect on feed intake can be made from the expected stimulation of DM intake by biotin supplementation as established by meta-analysis [39], whereas DM intake-increasing effects of PBLC have been observed only occasionally [63]. The intakes of concentrates were not different among the groups; however, as we were unable to record the intake of PMR in the present study, we cannot proof or disproof stimulation of feed intake. Nonetheless, lower mobilization of body fat, lower mobilization of lean body mass and increased feed intake can all be judged as positive effects of PBLC+B on postpartum metabolic health and appear to distinguish PBLC+B from MON. Monensin has a proven feed-depressing action [20, 56, 64], which partially counteracts its several positive influences on postpartum metabolic health. The positive influence of MON on postpartum BW has been quantified at 60 g/d in a meta-analysis [64], which appears consistent with the results of the present study, but is far less than that observed for PBLC+B under otherwise identical conditions.

One frequently used criterion for judging the effects of a treatment on dairy cow metabolism is milk performance. As expectable from meta-analysis [64], MON increased milk yield in Phase 3 of the study; however, milk components responded primarily to PBLC+B treatment. A trend for higher milk protein concentration was observed in the PBLC+B group on the first herd test day. Interestingly, an almost identical trend for an increase in milk protein concentration by 0.2% on the first (but not second) herd test day was observed in our preceding pilot study involving PBLC+B on another farm [32]. The latter greatly substantiates the present finding, despite the lack of statistical significance. Biotin is not known to affect milk protein concentration [39]. Therefore, the increase in milk protein concentration may be attributed either to the established effects of PBLC on milk protein output [31, 41] or to the combined effect of both PBLC and biotin.

Milk fat concentration responded to treatment on the second test day when PBLC+B cows showed higher values than MON cows, with CON cows being intermediate. A higher milk fat percentage of the PBLC+B group was also indicated as a trend from the daily estimates of the milking robot over the whole observation period (Phase 2 and Phase 3). Part of the contrast in the milk fat concentration of PBLC+B vs. MON cows is probably attributable to the milk fat-depressing action of MON. The latter has been linked to a higher concentration of *trans*-10 18:1 fatty acid in milk, indicative of the impaired bio-hydrogenation of linoleic acid in the rumen [54, 65]. *Trans*-10 18:1 fatty acid, on the other hand, is a potent inhibitor of milk fat synthesis [66]. Alternatively, a higher milk fat concentration could also, in part, result from enhanced milk fat synthesis during PBLC+B treatment. An increase in milk fat concentration is not typically observed when PBLC supplementation of cows is applied after the transition period [31, 65]. However, an increase in milk fat percentage has been seen in high-conditioned (BCS 3.95) transition cows with restricted concentrate feeding and PBLC supplementation, as reported by Drong et al. [21]. The latter authors suggested that this might be attributable to the enhanced clearance of circulating NEFA by the mammary gland in PBLC-supplemented cows. Biotin supplementation could theoretically also stimulate milk fat synthesis, although this is not typically seen in dairy cows [39]. However, a key role of biotin for fatty acid synthesis in the bovine mammary gland is deductible from the profound inhibition of mammary fatty acid synthesis by the biotin antagonist avidin [67], supporting the view that an increased supply of biotin as cofactor of acetyl-CoA synthase can enhance lipid generation from ruminally-produced acetate.

The differences in the milk fat concentration together with a milk yield intermediate between CON and MON resulted in increased yields of energy-corrected milk for PBLC+B vs. CON cows of ~3 to 4 kg/d in both treatment phases. Moreover, ECM yield was also higher in PBLC+B cows compared to MON cows in Phase 2. This conforms with results of previous studies with the used PBLC at dosages of 1 to 2 g/d per cow [32, 41, 63] and with results of a meta-analysis of 238 cows in 11 trials with biotin at dosages of 20 mg/d per cow, showing an increase in daily milk yield. Notably, this increase in ECM yield was associated with lower BW loss and was, as such, not produced at the expense of body condition.

## Conclusion

Negative energy balance and excessive BW loss are major challenges that coincide with subclinical ketosis and associated diseases in postpartum dairy cows. The key result of the present study is that a combination of PBLC+B prevents body weight loss after parturition almost completely. Future studies will have to elucidate to what extent this PBLC+B effect is attributable to a better conservation of body fat, a better conservation of lean body mass and/or an increased DM intake. Supplementation of PBLC+B were further associated with a similar ketosis incidence and partly higher yields of energy-corrected milk compared with MON-supplemented cows. Thus, the PBLC+B treatment appears to be an attractive alternative to MON supplementation in transition dairy cows, with a high potential of reducing antibiotic use in agriculture.

## Supporting information

S1 Table Postpartum concentrate intake, milk robot visits and milk yield. (PDF)

S2 Table Back fat thickness, body condition score and body weight. (PDF)

S3 Table Serum concentrations of key indicators of energy metabolism. (PDF)

S4 Table Serum concentrations of indicators of liver function. (PDF)

S5 Table Serum activities of enzymes. (PDF)

S1 Fig Change in body weight. (PDF)

S2 Fig Milk fat and protein percentages and energy-corrected milk yield derived from daily measurements of the milking robot. (PDF)
