# Supplementary material for: Effects of a combination of plant bioactive lipid compounds and biotin compared with monensin on body condition, energy metabolism and milk performance in transition dairy cows
Source: PLoS One. 2018 Mar 27;13(3):e0193685. doi: 10.1371/journal.pone.0193685 (PMC5870966; doi:10.1371/journal.pone.0193685)
Supplement: S1 Table — (PDF) [file pone.0193685.s001.pdf]

S1 Table. Postpartum concentrate intake, milk robot visits and milk yield of cows receiving plant bioactive lipid compounds and biotin (PBLC+B) from d -21 to d 37 relative to parturition, cows receiving a monensin bolus (MON) at d -21 or cows receiving no such supplements (CON)

Day	Concentrate[1] intake, kg/d					Milk robot visits per day					Milk yield, kg/d				
	CON	PBLC+B	MON	SEM	*P*-value	CON	PBLC+B	MON	SEM	*P*-value	CON	PBLC+B	MON	SEM	*P*-value
2	2.44	2.21	2.32	0.128	0.28	2.0	1.9	2.2	0.13	0.33	18.3	17.2	18.8	1.60	0.45
5	2.85	2.75	2.54	0.120	0.19	2.4	2.1	2.4	0.15	0.42	29.5	29.5	28.7	1.49	0.75
8	3.41	3.28	3.15	0.164	0.076	2.9	2.4	2.7	0.23	0.12	33.8	33.2	32.6	1.51	0.80
11	3.96	3.92	3.77	0.212	0.74	2.9	2.8	3.0	0.25	0.84	35.0	35.6	35.7	1.81	0.76
14	4.70	4.53	4.34	0.227	0.51	3.0	2.9	3.3	0.29	0.25	36.5	37.6	38.3	1.77	0.84
17	5.35	5.21	4.84	0.239	0.35	3.2	2.9	3.4	0.29	0.13	38.1	39.3	40.2	1.95	0.70
20	5.97	5.79	5.43	0.296	0.44	3.1	3.1	3.2	0.28	0.83	39.6	40.6	42.4	1.96	0.38
23	6.46	6.23	5.96	0.374	0.62	3.0	3.1	3.4	0.29	0.45	40.9	42.6	43.5	2.02	0.60
26	6.35	6.49	6.42	0.463	0.97	2.9	3.0	3.3	0.29	0.29	41.5	42.5	45.2	2.02	0.43
29	6.29	6.75	6.51	0.469	0.73	2.9	3.3	3.3	0.30	0.42	41.6	44.0	45.8	2.04	0.27
32	6.66	6.53	6.59	0.447	0.97	3.1	3.2	3.3	0.32	0.88	42.3	44.9	46.0	2.35	0.34
35	6.77	6.54	6.55	0.463	0.90	3.2	3.1	3.3	0.29	0.83	44.1	44.7	45.9	2.26	0.81
38	6.95	6.52	6.63	0.451	0.72	3.3	3.3	3.3	0.25	0.97	44.9	45.1	47.3	1.99	0.54
41	6.90	6.53	6.70	0.481	0.80	3.4	3.3	3.4	0.24	0.74	44.9	44.7	47.3	1.89	0.47
44	7.10	6.64	6.76	0.449	0.63	3.3	3.3	3.4	0.29	0.88	44.4	45.5	47.0	1.84	0.53
47	6.79	6.78	7.12	0.465	0.68	3.3	3.4	3.4	0.29	0.77	43.5	45.6	47.8	2.07	0.18
50	6.94	6.88	7.31	0.436	0.49	3.2	3.4	3.3	0.30	0.89	43.9	45.3	47.7	2.15	0.37
53	6.80	6.71	7.36	0.468	0.22	3.2	3.3	3.4	0.31	0.75	42.9	45.5	46.9	2.31	0.32
56	6.86	7.03	7.46	0.385	0.19	3.2	3.4	3.4	0.32	0.55	43.1	44.9	47.9	2.21	0.12

Data were pooled per cow over 3 consecutive days and are presented as means and pooled SEM of 17 cows in the CON group, 18 cows in the PBLC+B group and 18 cows in the MON group.

[1]Concentrates included a fixed allowance of 1 kg/d concentrate C1 (groups CON and MON) or C1*PBLC+B (group PBLC+B; for composition, see Table 1), which was delivered first at the first daily milking. The rest of the total concentrate allowance consisted of equal parts of C1 and C2.
