# Supplementary material for: Effects of a combination of plant bioactive lipid compounds and biotin compared with monensin on body condition, energy metabolism and milk performance in transition dairy cows
Source: PLoS One. 2018 Mar 27;13(3):e0193685. doi: 10.1371/journal.pone.0193685 (PMC5870966; doi:10.1371/journal.pone.0193685)
Supplement: S2 Table — (PDF) [file pone.0193685.s002.pdf]

S2 Table. Back fat thickness, body condition score and body weight of cows receiving plant bioactive lipid compounds and biotin (PBLC+B) from d -21 to 37 relative to parturition, cows receiving a monensin bolus (MON) at d -21 or cows receiving no such supplements (CON)

Day	Back fat thickness, cm					Body condition score					Body weight, kg				
	**CON**	**PBLC+B**	**MON**	**SEM**	***P*-value**	**CON**	**PBLC+B**	**MON**	**SEM**	***P*-value**	**CON**	**PBLC+B**	**MON**	**SEM**	***P*-value**
-21	2.86	3.29	3.11	0.355	0.24	3.57	3.82	3.71	0.236	0.47					
-7	2.67	3.29	3.00	0.358	0.45	3.51	3.67	3.50	0.222	0.21					
2	2.52	3.15	2.74	0.340	0.39	3.29	3.49	3.42	0.223	0.13	703	746	739	34.5	0.38
9	2.26	2.89	2.57	0.321	0.19	3.09	3.33	3.19	0.210	0.102	672	729	712	32.5	0.19
16	1.99	2.57	2.21	0.304	0.29	2.90	3.11	2.99	0.206	0.093	651^{b}	731^{a}	698^{ab}	28.0	0.025
23	1.70	2.27	1.96	0.294	0.25	2.78	3.00	2.86	0.198	0.080	657	723	698	28.4	0.072
30	1.54	2.03	1.85	0.265	0.45	2.69	2.86	2.81	0.198	0.16	653^{b}	730^{a}	694^{ab}	29.1	0.036
37	1.40	1.93	1.66	0.261	0.15	2.56	2.79	2.69	0.149	0.12	646^{b}	731^{a}	692^{ab}	29.4	0.020
44	1.26	1.77	1.55	0.249	0.30	2.51^{b}	2.69^{a}	2.64^{ab}	0.186	0.030	649^{b}	732^{a}	688^{ab}	27.9	0.007
51	1.18	1.64	1.49	0.249	0.25	2.47	2.67	2.61	0.187	0.065	656^{b}	748^{a}	712^{ab}	28.7	0.008
58	1.09	1.53	1.44	0.242	0.12	2.37	2.61	2.57	0.199	0.061	656^{b}	747^{a}	714^{b}	28.1	0.005

Data are means and pooled SEM of 17 cows in the CON group, 18 cows in the PBLC+B group and 18 cows in the MON group. Body weight data were pooled over three consecutive days per animal before statistical analysis.

^{ab}Superscript letters indicate differences among treatment groups at $P < 0.05$.
