# Supplementary material for: Effects of a combination of plant bioactive lipid compounds and biotin compared with monensin on body condition, energy metabolism and milk performance in transition dairy cows
Source: PLoS One. 2018 Mar 27;13(3):e0193685. doi: 10.1371/journal.pone.0193685 (PMC5870966; doi:10.1371/journal.pone.0193685)
Supplement: S4 Table — (PDF) [file pone.0193685.s004.pdf]

S4 Table. Serum concentrations of indicators of liver function in cows receiving plant bioactive lipid compounds and biotin (PBLC+B) from d -21 to 37 relative to parturition, cows receiving a monensin bolus (MON) at d -21 or cows receiving no such supplements (CON)

Day	Cholesterol, mmol/L					Bilirubin, µmol/L					Urea, mmol/L				
	**CON**	**PBLC+B**	**MON**	**SEM**	***P*-value**	**CON**	**PBLC+B**	**MON**	**SEM**	***P*-value**	**CON**	**PBLC+B**	**MON**	**SEM**	***P*-value**
-21	2.85	2.84	2.84	0.268	1.00	0.64	0.61	0.63	0.167	0.66	4.37	4.31	4.36	0.261	0.97
-7	2.05	2.02	2.05	0.170	0.98	1.39	1.33	1.65	0.468	0.78	3.94	4.32	4.32	0.259	0.23
2	1.65	1.61	1.73	0.144	0.74	5.05	3.91	5.94	1.206	0.74	4.35	4.53	4.89	0.378	0.77
9	2.19	2.10	2.05	0.199	0.75	3.24	2.79	3.51	0.783	0.94	4.14	3.92	4.43	0.268	0.27
16	2.70	2.80	2.63	0.244	0.76	2.62	1.68	2.06	0.644	0.75	4.28	3.86	4.62	0.305	0.054
23	3.24	3.51	3.33	0.288	0.60	1.37	1.24	1.30	0.351	1.00	4.48	4.48	4.92	0.341	0.33
30	3.71	3.92	3.99	0.299	0.59	1.08	0.84	0.83	0.281	0.63	4.63	4.39	4.93	0.303	0.21
37	4.02	4.35	4.44	0.334	0.41	0.81	0.83	0.84	0.240	0.83	5.20	4.72	5.12	0.300	0.24
44	4.40	4.71	4.85	0.346	0.41	0.56	0.90	0.81	0.235	0.57	4.94	4.91	5.15	0.239	0.76
51	4.64	4.93	5.05	0.356	0.46	0.67	0.77	0.50	0.187	0.43	5.18	4.94	5.38	0.308	0.37
58	4.80	4.96	5.09	0.380	0.74	0.51	0.53	0.50	0.120	0.95	5.48	4.95	5.58	0.271	0.063

Data are means and pooled SEM of 17 cows in the CON group, 18 cows in the PBLC+B group and 18 cows in the MON group.
