# Supplementary material for: Effects of a combination of plant bioactive lipid compounds and biotin compared with monensin on body condition, energy metabolism and milk performance in transition dairy cows
Source: PLoS One. 2018 Mar 27;13(3):e0193685. doi: 10.1371/journal.pone.0193685 (PMC5870966; doi:10.1371/journal.pone.0193685)
Supplement: S5 Table — (PDF) [file pone.0193685.s005.pdf]

S5 Table. Serum activities of enzymes in cows receiving plant bioactive lipid compounds and biotin (PBLC+B) from d -21 to 37 relative to parturition, cows receiving a monensin bolus (MON) at d -21 or cows receiving no such supplements (CON)

Day	Aspartate transaminase, U/L					γ-Glutamyltranspeptidase, U/L					Glutamate dehydrogenase, U/L				
	CON	PBLC+B	MON	SEM	*P*-value	CON	PBLC+B	MON	SEM	*P*-value	CON	PBLC+B	MON	SEM	*P*-value
-21	70.5	70.7	77.6	6.01	0.82	25.1	26.2	27.5	2.24	0.61	13.8	14.1	13.7	4.04	0.73
-7	66.5	64.4	82.4	4.91	0.17	22.4	23.5	24.5	1.75	0.62	11.0	9.9	15.4	2.39	0.23
2	96.5	94.4	107.6	9.96	0.49	22.1	21.8	24.4	1.54	0.96	9.2	7.3	10.6	1.67	0.36
9	109.6	104.2	126.6	13.66	0.40	24.6	23.6	26.4	1.54	0.30	13.6	14.4	17.3	4.45	0.14
16	103.5	95.0	109.0	8.90	0.60	26.8	26.9	30.6	2.37	0.14	24.9	20.5	32.9	8.18	0.14
23	91.4	84.5	94.6	6.77	0.64	29.6	29.8	35.5	3.05	0.26	26.1	23.3	38.4	9.48	0.46
30	91.3	74.4	87.3	7.43	0.13	35.7	30.2	40.9	6.03	0.50	31.7	19.8	42.0	11.77	0.34
37	82.3	73.2	112.8	5.25	0.68	31.8	30.4	39.9	3.50	0.36	21.4	21.9	28.8	9.62	0.71
44	78.4	71.3	83.8	5.00	0.69	31.1	30.5	38.1	2.67	0.25	19.0	18.8	19.6	7.34	0.71
51	79.0	71.1	79.1	6.01	0.40	30.3	30.5	35.7	2.26	0.32	17.0	15.3	21.1	4.17	0.56
58	84.3	68.8	80.9	6.83	0.096	29.8	29.5	33.9	1.81	0.37	22.0	13.6	16.2	5.21	0.44

Data are means and pooled SEM of 17 cows in the CON group, 18 cows in the PBLC+B group and 18 cows in the MON group.
