# Supplementary material for: Effects of a combination of plant bioactive lipid compounds and biotin compared with monensin on body condition, energy metabolism and milk performance in transition dairy cows
Source: PLoS One. 2018 Mar 27;13(3):e0193685. doi: 10.1371/journal.pone.0193685 (PMC5870966; doi:10.1371/journal.pone.0193685)
Supplement: S1 Fig — (PDF) [file pone.0193685.s006.pdf]

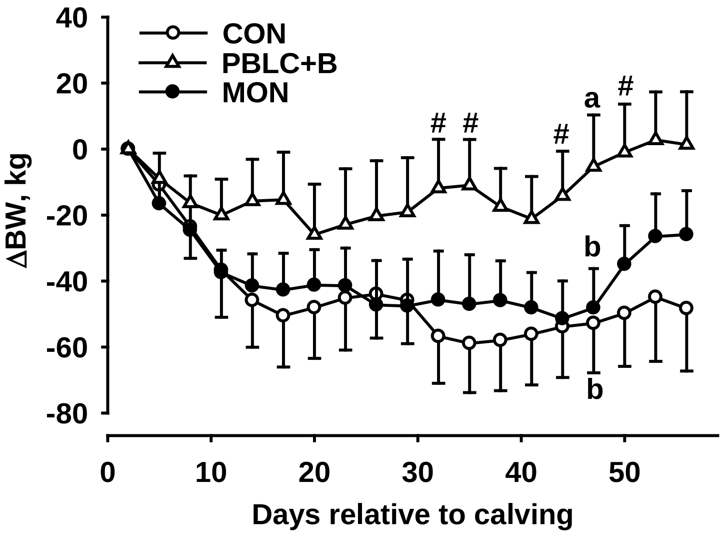

S1 Figure. Change in body weight (ΔBW) of cows supplemented with 2 g/d plant bioactive lipid compounds and 40 mg/d biotin (PBLC+B) between d -21 and d 37 in comparison with cows receiving a commercial monensin bolus (MON) at d -21 and control (CON) cows without such supplements. Data are means with SEM of 17 cows in the CON group, 18 cows in the PBLC+B group and 18 cows in the MON group. Body weight data were pooled over three consecutive days per animal before statistical analysis by one-way ANOVA at each individual time point. As body weight was recorded by the milking robot, d 2 (i.e. the arithmetic mean of d 1 to d 3) after calving was the first available value and was used as a reference to calculate ΔBW for each cow. $^{\#}P < 0.1$; $^{a,b}P < 0.05$.
