# Supplementary material for: Effects of a combination of plant bioactive lipid compounds and biotin compared with monensin on body condition, energy metabolism and milk performance in transition dairy cows
Source: PLoS One. 2018 Mar 27;13(3):e0193685. doi: 10.1371/journal.pone.0193685 (PMC5870966; doi:10.1371/journal.pone.0193685)
Supplement: S2 Fig — (PDF) [file pone.0193685.s007.pdf]

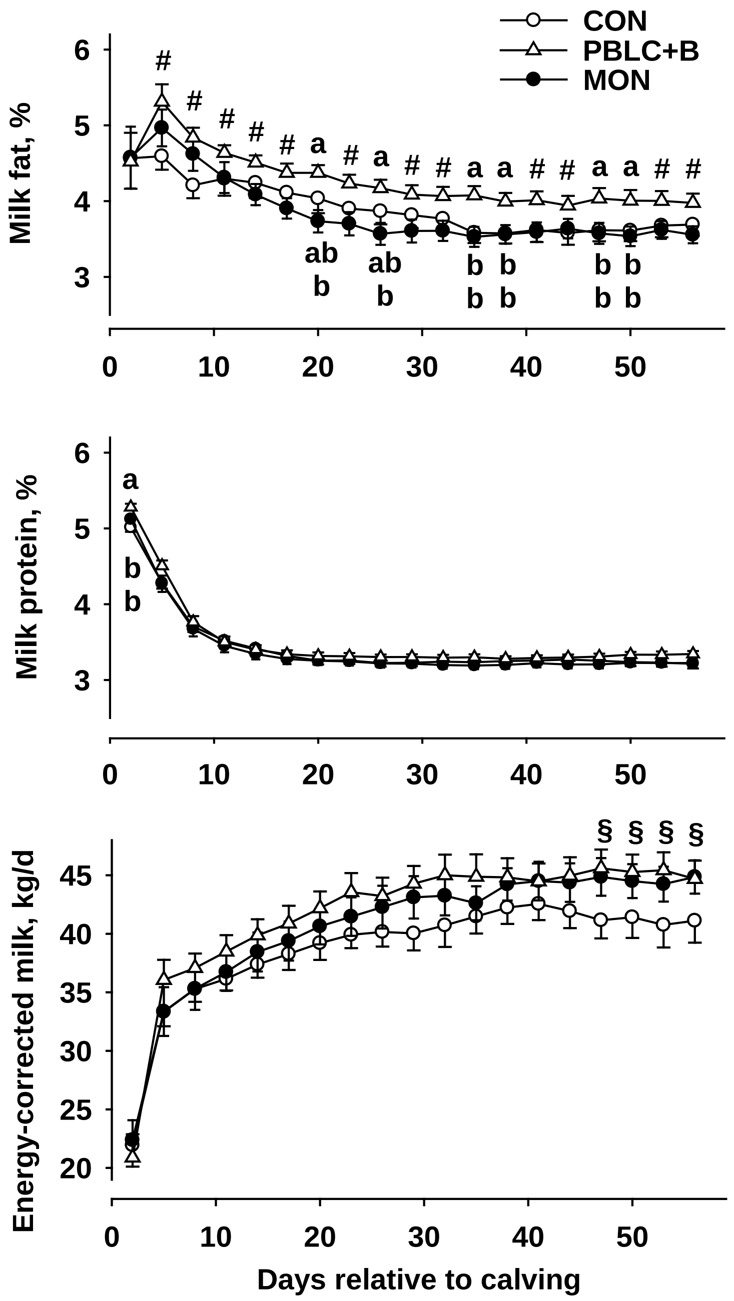

S2 Figure. Milk fat and protein percentages and energy-corrected milk yield (ECM) derived from daily measurements of the milking robot. Cows were supplemented with 2 g/d plant bioactive lipid compounds and 40 mg/d biotin (PBLC+B) between d -21 and d 37 and compared with cows receiving a commercial monensin bolus (MON) at d -21 and control (CON) cows without either supplement. Data are means with SEM of 17 cows in the CON group, 18 cows in the PBLC+B group and 18 cows in the MON group. Energy-corrected milk yield was calculated by using the formula ECM (kg/d) = milk yield (kg/d) × [0.038 × fat (g/kg) + 0.021 × protein (g/kg) + 1.05] / 3.28. Data were pooled over three consecutive days per animal before statistical analysis by one-way ANOVA at each individual time point. $^{\#}P < 0.1$; $^{a,b}P < 0.05$.
